# Entropy Change of Biological Dynamics in Asthmatic Patients and Its Diagnostic Value in Individualized Treatment: A Systematic Review

**DOI:** 10.3390/e20060402

**Published:** 2018-05-24

**Authors:** Shixue Sun, Yu Jin, Chang Chen, Baoqing Sun, Zhixin Cao, Iek Long Lo, Qi Zhao, Jun Zheng, Yan Shi, Xiaohua Douglas Zhang

**Affiliations:** 1 Faculty of Health Sciences, University of Macau, Taipa, Macau, China; 2 State Key Laboratory of Respiratory Disease, the 1st Affiliated Hospital of Guangzhou Medical University, Guangzhou 510230, China; 3 Beijing Engineering Research Center of Diagnosis and Treatment of Respiratory and Critical Care Medicine, Beijing Chaoyang Hospital, Beijing 100043, China; 4 Department of Geriatrics, Centro Hospital Conde de Sao Januario, Macau, China; 5 Department of Mechanical and Electronic Engineering, Beihang University, Beijing 100191, China

**Keywords:** entropy, irregularity, asthma, physiological signal, individualized treatment

## Abstract

Asthma is a chronic respiratory disease featured with unpredictable flare-ups, for which continuous lung function monitoring is the key for symptoms control. To find new indices to individually classify severity and predict disease prognosis, continuous physiological data collected from monitoring devices is being studied from different perspectives. Entropy, as an analysis method for quantifying the inner irregularity of data, has been widely applied in physiological signals. However, based on our knowledge, there is no such study to summarize the complexity differences of various physiological signals in asthmatic patients. Therefore, we organized a systematic review to summarize the complexity differences of important signals in patients with asthma. We searched several medical databases and systematically reviewed existing asthma clinical trials in which entropy changes in physiological signals were studied. As a conclusion, we find that, for airflow, heart rate variability, center of pressure and respiratory impedance, their entropy values decrease significantly in asthma patients compared to those of healthy people, while, for respiratory sound and airway resistance, their entropy values increase along with the progression of asthma. Entropy of some signals, such as respiratory inter-breath interval, shows strong potential as novel indices of asthma severity. These results will give valuable guidance for the utilization of entropy in physiological signals. Furthermore, these results should promote the development of management and diagnosis of asthma using continuous monitoring data in the future.

## 1. Introduction

Asthma is a chronic respiratory disease with an increasing incident rate (8% in 2009 vs. 7% in 2001, globally) and its typical symptoms include breathing constriction, reduced oxygen intake, limitation of activity, and even life-threatening respiratory failures that require immediate intervention [1]. It is reported that asthma affects 234 million people around the world and places a heavy burden on patients and their families [2]. The foremost purposes of asthma therapy are to achieve and maintain the control of symptoms, reduce respiratory impairments, and prevent exacerbation [3]. Appropriate management of asthma can help patients live a relatively high quality of life [2]. The lung function is the key indicator of potential risk assessment, thus continuous monitoring of lung function plays a pivotal role in the treatment and care of asthmatic patients.

Besides the classical measurement of static lung function tested at particular time points, increasing attention is being paid to the study of pulmonary function dynamics in expectation of discovering novel indices that can reflect and predict fluctuation of respiratory function during a longer period [4,5]. For this reason, nonlinear analysis methods are widely used to study the complexity of biological data collected from continuous monitoring devices. One of the meaningful characteristics of these dynamic biological data is the irregularity measured by entropy. Currently, several extended concepts of entropy, such as approximate entropy (ApEn) [6], cross-approximate entropy (Cross-ApEn) [7], sample entropy (SampEn) [8] and multi-scale entropy [9,10] are being applied in the quantification of inner irregularity of physiological signals and classification of different disease phases or severities. Entropy has been applied in the classification of different biological parameters of various diseases [10,11,12]. Research articles have been published on the patterns of entropy change of electroencephalogram (EEG) or electrocardiogram (ECG) signals of epilepsy [13], ECG segments or sound signals of obstructive sleep apnea syndrome, heart rate variability (HRV) of cardiovascular diseases, respiratory signals in chronic obstructive pulmonary disease (COPD), and so on [11,14,15,16].

In the area of asthma research, several clinical trials investigating the entropy of various physiological signals associated with asthmatic disease have already been carried out [17,18,19,20,21,22,23,24]. These signals include airflow, heart rate variability, center of pressure and respiratory impedance, respiratory sound, airway resistance, and so on. Do all these signals have the same trend of changes in entropy? In what types of signals does entropy show high potential as novel indices of asthma severity? To answer these questions, a systematic review on all available key literatures in this field has yet to be conducted. Therefore, we present this systematic review in which we obtain the overall trend of entropy change in the physiological signals related to asthma, evaluate the potential of using entropy of biological dynamics as new clinical indices and discuss possible strategies of future research. These results will give valuable guidance for the utilization of entropy in physiological signals measured by health monitoring devices as well as for research on complexity in asthma. Furthermore, these results should promote the development of management and diagnosis of asthma in the future.

## 2. Materials and Methods

### 2.1. Search Strategy and Inclusion Criteria

**Figure 1:**
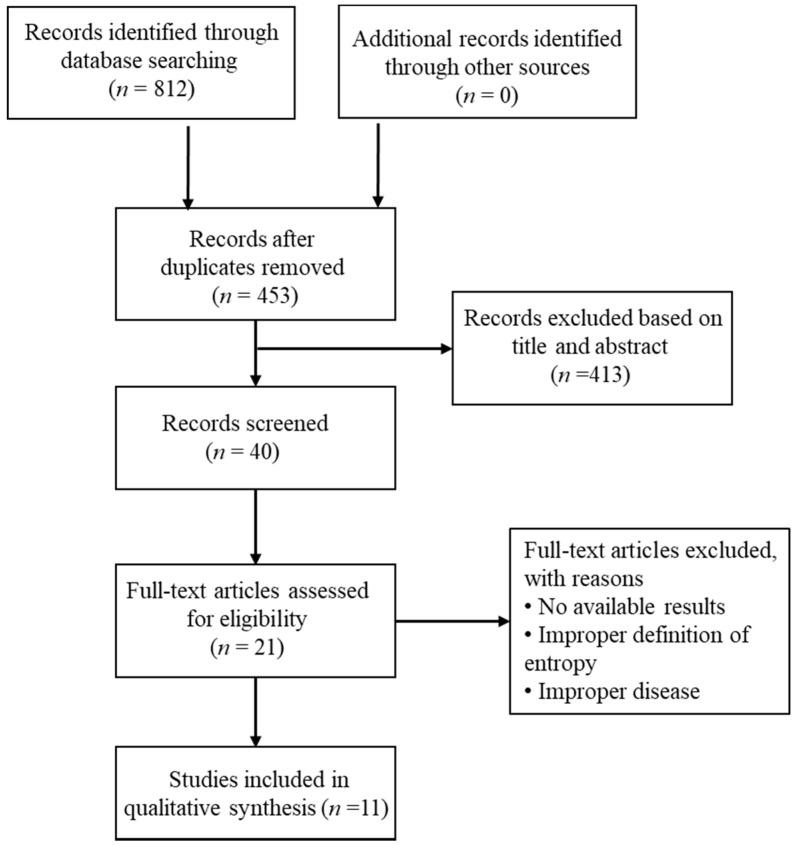
PRISMA flow diagram.

Databases of EMBASE, PubMed, and Google Scholar were searched for papers on the application of entropy analysis in asthma until May 2017. Key words used for our search included “asthma”, “asthmatic”, “respiratory tract diseases”, “respiratory disorders”, and “entropy”. The search in these databases resulted in 812 articles and 40 articles remained after removal of duplicates and exclusion of records based on title and abstract. The Preferred Reporting Items for Systematic Reviews and Meta-Analyses (PRISMA) workflow is shown in Figure 1.

Trials included in the final discussion met the following criteria: (1) patients with asthma were studied; (2) entropy of asthma associated physiological signals was analyzed; and (3) study results were published. Conference abstracts were excluded for the uncertainty of their reliability as they had not been strictly peer-reviewed. After the search, 11 trials meeting the criteria were reviewed.

### 2.2. Information Extraction and Quality Evaluation

The following information, if possible, was gathered from the reviewed studies: study type, study design, subject number, age, gender ratio, pulmonary function, physiologic signals, entropy types and results.

Jadad’s GRADE (grading of recommendations assessment, development, and evaluation) scale was used to evaluate the quality of the publications [25]. Overall quality of the articles was assessed based on their study type, study design (randomization and blinding methods), and description of participants’ outcome. The strength of the recommendation to conduct our systematic analysis using these studies was finally graded as very low, low, moderate, or high (Table 1).

### 2.3. Brief Introduction of Entropies

The concept of entropy was proposed for the first time in 1865 by Rudolf Clausius. Physicist Ludwig Boltzmann deduced the physical nature of entropy which can be associated with internal disorder in 1870. In 1948, Shannon extended the concept of entropy in statistical physics to the process of channel communication, thus creating the discipline “information theory”. Since Shannon’s extension of the initial thermodynamic concept of entropy, the application of entropy was gradually expanded to the area of statistics [26], information theory [27,28], physical system [29,30], and then physiological dynamic system.

Shannon’s definition of “entropy” is also known as “Shannon entropy” or “information entropy” [31], which is calculated as:

$$S\left(p_{1},p_{2},\ldots ,p_{n}\right)=\;-K\overset{n}{\underset{i=1}{\sum }}\left(p_{i}\log p_{i}\right)$$

In the formula, *i* stands for all possible samples in the probability space, *p_i_* is the probability of occurrence of the sample, and *K* is a constant. Kolmogorov entropy calculates the change rate of Shannon entropy of a system. The calculation of Kolmogorov entropy is rather complicated. Thus, the lower bound of Kolmogorov entropy is commonly used [28]. For the analysis of short and noisy time series, Pincus [6] introduced a family of measures termed approximate entropy as approximate estimation to the lower bound of Kolmogorov entropy. Then, sample entropy was introduced to eliminate self-matches effect in approximate entropy. Sample entropy [8] has the advantage of being less dependent on time series length and showing relative consistency over a broader range of parameter values. In the 2000s, the multiscale entropy (MSE) was promoted by Costa et al. [10] to represent the complexity of a signal in different time scales. MSE relies on the computation of the sample entropy over a range of scales. In the MSE algorithm, coarse-grained time series, which represent the system dynamics on different scales, are analyzed with the sample entropy algorithm. For scale one, the MSE value of a time series corresponds to the sample entropy value.

## 3. Results

In the studies resulted from our database search, various asthma associated physiological signals have been collected and analyzed. The signals we studied include airflow, heart rate variability, center of pressure, respiratory impedance, airway resistance, respiratory sound, etc. (Table 2).

### 3.1. Airflow

The pathophysiology of asthma is characterized by chronic airway obstruction which gradually affects the entire tracheobronchial tree. Therefore, asthma patients may have altered airflow pattern, control of respiration, and entropy in respiratory dynamics [25]. There are studies aiming to characterize the complexity of respiratory patterns using nonlinear dynamical analysis [32,33,34].

Veiga et al. [21] conducted an observational clinical trial on the relationship between increased airway obstruction and changes of ApEn in the airflow dynamics of asthmatic patients. Totally, 26 subjects were involved in this study: 5 healthy subjects compared with 21 asthmatics patients with different levels of airway obstruction (5 normal spirometry exam (NE), 5 mild, 6 moderate, and 5 severe). It was found that there was a significant decrease in asthmatic patients in ApEn of airflow (*p* < 0.002) which was significantly correlated with the airway obstruction indices of Forced Expiratory Volume in the first second (FEV1) (R = 0.60; *p* < 0.001). Viega et al. then conducted an enlarged study [35] with 62 subjects: 11 healthy controls and 51 asthmatic patients with different levels of airway obstruction (11 NE, 14 mild, 14 moderate, and 12 severe). Significant decrease of ApEn was observed in asthma groups (*p* < 0.02) which was significantly correlated with FEV1 (%) (R = 0.31; *p* = 0.013). Receiver Operating Characteristic (ROC) curve was plotted, which shows that the area under the curve (AUC) for ApEn of airflow reached acceptable values for clinical use in discriminating healthy subjects from patients with mild, moderate, and severe airway obstruction (AUC > 0.8).

Raoufy et al. [36] used four nonlinear analysis methods (sample entropy, cross-sample entropy, largest Lyapunov exponents, and detrended fluctuation analysis) to compare the respiratory complexity pattern in asthmatic patients with that of healthy subjects. Forty subjects were enrolled in this study: 10 healthy subjects and 30 patients with different types of asthma (controlled atopic asthma (CAA), uncontrolled atopic asthma (UAA), and uncontrolled non-atopic asthma (UNAA), with 10 patients for each group). The metrics of inter-breath interval (IBI) and lung volume (LV) were continuously recorded and analyzed using nonlinear analysis methods. Significant decrease of SampEn in IBI (*p* < 0.001) and LV (*p* = 0.002) was observed in the asthma group than in the healthy control, while no significant difference was found in SampEn of LV between the patients in UNAA group and healthy subjects. Cross-sample entropy between IBI and LV was further calculated, which showed increased synchronization in UAA and UNAA groups (*p* < 0.001). ROC curve was drawn and SampEn showed a high clinical potential as a complexity index in differentiating asthmatic patients from healthy people (AUC = 0.95, 95% CI: 0.89–1.02).

### 3.2. Heart Rate Variability

Cardiac function is often highly influenced by breath difficulty in asthma patients. The increased obstruction during respiration gradually causes imbalance in the autonomic regularity of heart rhythm. One clinic index used to measure the variation of cardiac function is heart rate variability (HRV) which assesses the variation in the R-wave intervals.

Garcia-Araujo et al. [19] studied the HRV in the supine, seated positions and during the respiratory sinus arrhythmia maneuver (M-RSA) in 14 asthmatic and 10 healthy subjects. Nonlinear analysis methods including ApEn, SampEn, and Shannon entropy were used to evaluate the complexity/irregularity of HRV. It was found that, in the asthma group, the patients had a higher SampEn in the supine position than in the seated position (*p* < 0.05). During the M-RSA, a significantly lower value (*p* < 0.05) of ApEn was observed in asthma group than that in control group. The correlation between airway obstruction and HRV entropies also showed potential as biological markers of cardiovascular function impairment due to asthma.

### 3.3. Center of Pressure

Long-term asthma may lead to alteration in motor function and decrease in postural control. Postural control is clinically evaluated by the magnitude of variability of the center of pressure (COP) during standing in different conditions. Kuznetsov et al. [37] conducted research to investigate the ability of postural control in 21 young asthma patients and 18 age-matched control participants. The results showed no significant difference in the task completion between asthma and control group, while SampEn of COP_AP_ (anterior-posterior COP data, collected by a Bertec force plate) was lower in the asthma group (*p* = 0.04).

### 3.4. Respiratory Sound

The respiratory sound signals of asthmatic patients carry important information might be used for discriminating different disease stages. The complexities of respiratory sound signals are higher in patients with lung function impairment than in healthy people because of the existence of additional auxiliary signals in unhealthy lungs. However, the complexity of respiratory sound dynamics makes it difficult to access the disease status using the baseline entropy measures. Recently, some novel algorithms have been proposed to improve the accuracy and sensitivity in distinguishing different types of respiratory sounds.

Jin et al. [38] conducted an observational study to test a novel statistical algorithm. It uses SampEn histograms of the respiratory sound signals to identify wheeze segments, a type of lung sound featured with periodic waveforms at a dominant frequency above 100 Hz and with a duration of over 100 ms. The sound signal recordings were collected from seven healthy and seven asthmatic subjects as well as from two lung sounds databases. Sample entropy of the sound data was calculated followed by mapping a histogram of mean distortion of the SampEn. This algorithm had an overall accuracy of 97.9% when detecting high-intensity wheezes and an accuracy of 85.3% when identifying low-intensity wheezes.

Aydore et al. [24] tested their algorithm for dividing wheeze and non-wheeze segments within respiratory sounds. The respiratory sound signal recoding from patients with asthma and COPD was collected in hospital’s database. The sound signals were divided into wheeze and non-wheeze segments based on the calculation of their four features: kurtosis, Renyi entropy, f50/f90 ratio and mean-crossing irregularity. Then, all data were projected into four-dimensional feature space applying Fisher Discriminant Analysis and then was tested using Neyman–Pearson hypothesis. In the training dataset, their method had a final classification rate of 95.1% and, in the testing dataset, leave-one-out approach of the method resulted a correction rate of 93.5%.

Mondal proposed and tested a new classifier consisting of four statistical parameters: kurtosis, skewness, lacunarity, and sample entropy [24,39]. The experimental sound data, which was collected from various resources, contained 120 cycles of recordings from 10 normal and 20 abnormal individuals. The pathological problems being involved in this experiment contained asthma, COPD, and interstitial lung disease (ILD). Extreme learning machine and support vector machine networks were used to evaluate the efficiency of this method on discriminating abnormal sound signals from normal ones. The results, which were five-fold cross-validated, showed that the accuracy, sensitivity and specificity of this algorithm were 92.86%, 86.30%, and 86.90%, respectively.

### 3.5. Respiratory Impedance and Airway Resistance

The increase in the airflow obstruction in patients with asthma causes modification of system resistance during respiration. The forced oscillation technique (FOT) is the most commonly used method in clinic to collect physiology metrics of system impedance in respiratory tracts, such as airway resistance, airway reactance, and respiratory impendence (Zrs) [40]. It should be noted that there is relationship between airway resistance and respiratory impedance as described below. FOT applies external force oscillations on the spontaneous breathing, measures the pressure–flow patterns and determines lung mechanical parameters. Que et al. revealed that when the force oscillation has a frequency near 5 Hz, the impedance has a resonance and the magnitude of system impedance is approximately equal to the airway resistance [4]. Impulse oscillometry is a kind of FOT which utilizes impulse-shaped external pressure oscillation to estimate the airway resistance or the real part of respiratory impedance. The detected resistance is actually a component of the pressure drop over the entire respiratory system in phase with flow and normalized with flow at certain frequencies. Because the chest wall also has resistance, technically impulse oscillometry measures total respiratory resistance instead of airway resistance. However, when oscillation frequency is above 5 Hz, most of the detected resistance comes from the airways.

Veiga et al. [22] conducted a trial to study the relationship among the severity of airway obstruction in asthmatic patients, their approximate entropy of respiratory impedance (ApEnZrs) and entropy of the density of respiratory impedance recurrence period (RPDEnZrs). Totally 74 subjects were enrolled including 12 healthy subjects and 62 asthmatic patients with no (NE, *n* = 12), mild (*n* = 20), moderate (*n* = 18), and severe (*n* = 12) airway obstruction. Respiratory impedance was measured at 5 Hz using forced oscillation technique. The resulting signals of pressure (P) and airflow (Q) were used to calculate the respiratory impedance (Zrs = P/Q). It was found that both ApEnZrs and RPDEnZrs decreased significantly (*p* < 0.002 and *p* < 0.00001, respectively) with the increase of airway obstruction. The association was accessed using Pearson’s correlation which showed that ApEnZrs and RPDEnZrs presented inverse correlation with Zrs (*p* < 0.0001). ROC curve indicates that ApEnZrs presents AUCs < 0.80 in all the study groups while RPDEnZrs reached acceptable value for diagnostic use (AUCs > 0.80).

Gonem et al. [20] studied the pattern of SampEn in airway resistance of asthmatic patients and its correlation with the frequency of asthma symptom exacerbation. This study involved 66 patients with severe asthma (33 on Global Initiative for Asthma (GINA) treatment step 4 and 33 on GINA treatment step 5) and 30 healthy people with matched demographics. A Jaeger MasterScreen Impulse Osillometry (IOS) system was used to record airway resistance and reactance in a frequency range of 5–35 Hz. Data were collected both at baseline and following bronchodilator administration. The results showed that, compared with healthy subjects, asthmatic patients had increased SampEn of airway impedance. For example, median SampEn of reactance area at baseline was 0.42 in healthy group, compared to 1.05 in GINA 4 group and 1.19 in GINA 5 group (*p* < 0.0001). The results also showed that, among all the 6 parameters studied in this trial, SampEn of R5–R20 (resistance at 5 Hz minus that at 20 Hz, a measure of the diversity of bronchial tree obstruction) was the only variable independently associated with frequent exacerbations (defined as two or more exacerbations in the previous year) (*p* = 0.016, odds ratio = 3.23:0.1).

Umar et al. [41] investigated entropy changes in airway resistance of 66 patients with severe asthma (GINA Stage 4/5) and 27 controls. IOS system was used to measure the airway resistance in the 5–35 Hz range, both at baseline and after salbutamol inhalation. The results showed that the SampEn of airway resistance in severe asthma group increased significantly compared to control group (*p* = 0.007 and 0.009 at baseline and post bronchodilation, respectively), and was correlated significantly with the exacerbation frequency (r score = 0.3 both at baseline and post bronchodilation).

### 3.6. Summary

**Figure 2:**
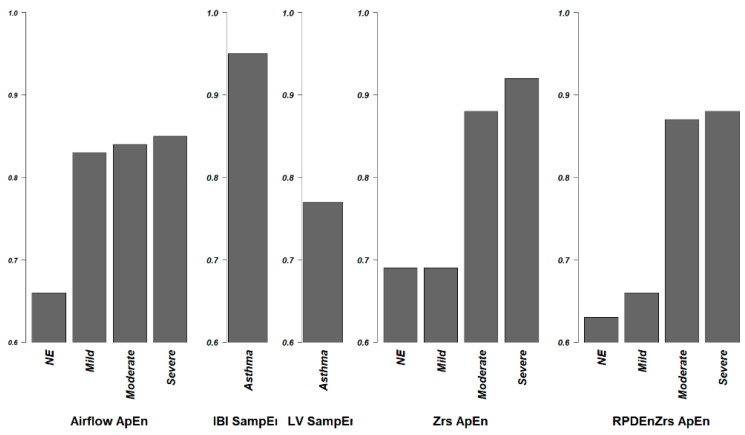
AUC area of Receiver Operating Characteristic curve. AUCs of some trials were calculated to evaluate the diagnostic ability of entropy for the severity of asthma. An index has an AUC value over 0.8 is considered good enough, and a value over 0.9 is considered excellent. SampEn of IBI has an excellent performance in distinguishing asthma patients from healthy people, ApEnZrs also performs excellently in distinguishing severe asthma patients. Besides, the ApEn of airflow has a good performance when distinguishing healthy subjects from patient with mild, moderate or severe asthma, ApEnZrs has a good performance in identifying moderate asthma patient, RPDEnZrs also performs well in distinguishing patients with moderate and severe asthma.

**Figure 3:**
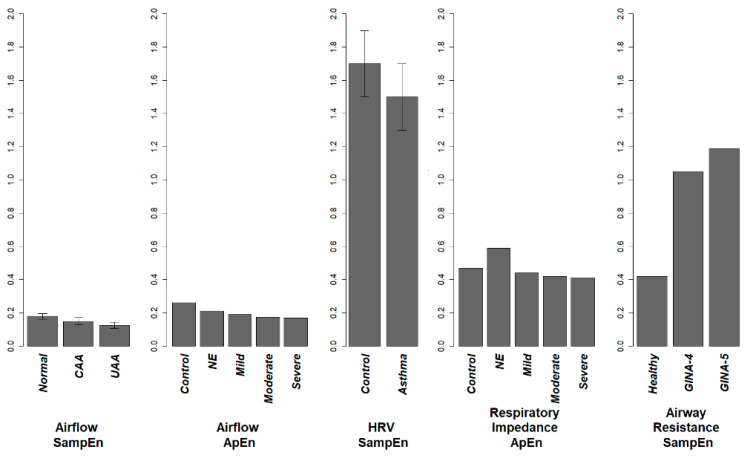
Complexity change in asthma. Entropy results are extracted and drawn to the same scale. Entropies of airflow, HRV and respiratory system impedance decrease associated with the disease progression. On the contrary, SampEn of airway impedance had a 2–3-fold increase in severe asthma patients. This contradiction might be explained by the way in which airway resistance is measured.

For the airflow, respiratory system impedance, heart rate, and center of pressure, the entropy of these signals are lower in an asthmatic patient when compared with a healthy subject. The loss of entropy in airflow, HRV, and respiratory impedance associate with the increase in airway obstruction. ApEn and SampEn of Airflow, and RPDEnZrs have shown acceptable potential as clinical indices when evaluated using the ROC curve (AUC area > 0.8) (Figure 2). For the respiratory sound and airway resistance and reactance, the entropy increases in asthmatic subjects. This general result discussed here is illustrated in Figure 3.

## 4. Discussion

With the rapid development of large data storage technologies and mobile network technologies, continuous monitoring of physiological signals is becoming increasingly important in the diagnosis and treatment of asthmatic patients [42,43]. Based on the continuous monitoring data, scientists hope to establish novel clinical indices for diagnosis and prognosis of asthma. Different from existing clinical examinations, these new indices will be able to classify patients into more precise disease types, so that more individualized treatment can be prescribed. Furthermore, the trends of some of these indices fluctuate prior to that of asthma symptoms, so that prevention can be addressed to avoid possible exacerbation.

Nonlinear analysis methods have been introduced to study the characteristics of these continuous dynamic data [39,40]. Recently, several studies have been conducted to explore the regularity of physiological signals in asthmatic patients [21,35,36,37]. To explore the pattern of complexity changes in asthma related physiological signals, we conducted this systematic review on available literature articles.

The quantified complexity of physiological signals obtained by entropy analysis provided novel perspective on the changes in asthma pathophysiology. Moreover, this review may contribute to the clinical evaluation of asthmatic patients and prediction of disease progression. From the summarized results, we can see that the pattern of entropy change in asthma may differs in different physiological signals. The entropy of physiological signals of HRV, airflow, center of pressure, and respiratory system impendence (Zrs = P/Q) are lower in a patient than in a healthy person, whereas the entropies of respiratory sound, airway resistance and reactance are higher in patients than in healthy people (Table 2).

The patterns of entropy in the physiological signals of airflow, HRV, and respiratory system impendence (Zrs) are similar to the results in previous studies on cardiovascular and COPD patients. This can be well explained by the widely accepted hypothesis proposed by Goldberger [44], who considered complexity as a kind of variability that “generates scale-invariance (self-similarity) and long-range organization”, said that the disease states are correspondingly characterized by a “loss of complexity”. The physiological signals of airway resistance and reactance, although detected in a similar method as that for respiratory system resistance, had an opposite patterns of entropy change. The reason for this inconsistency is currently unclear, but there may be several possible contributing factors. Firs, the method of impulse oscillometry in the published study is not as reliable as the standard forced oscillation technique using sinusoids. Second, the airway resistance is calculated differently from the impedance of entire respiratory system. As to respiratory sounds, the higher complexity in asthmatic subjects may be explained by the inclusion of sound signals which are more complex or heterogeneous in nature, such as wheeze or crackle. These highly irregular sound signals are products of the impaired respiratory tracts associated with the asthma progression [39].

ROC analysis is performed in some of these trials. The area under ROC is an index commonly used to evaluate whether the sensitivity and specificity of a new index are high enough to ensure reasonable type 1 and 2 error rates. Sample entropy of IBI has the highest AUC of 0.95 when comparing asthma and healthy subjects, however it did not have a high enough score when discriminating controlled from non-controlled asthma patients, or non-atopic from atopic asthma (AUC < 0.8). Approximate entropy of airflow, ApEnZrs, and RPDEnZrs had acceptable AUC values when comparing healthy subjects to asthma patient with moderate or severe airway obstruction. ApEn of airflow had an AUC > 0.8 when discriminating patient with mild airway obstruction to healthy people. None of the indices have an acceptable AUC value when comparing healthy subjects to asthmatic patient with normal spirometry exam. This is reasonable because, by definition, NE patients have normal lung function test; thus, the complexity of their biological dynamics is still unaffected.

Although the entropy of respiratory sounds is generally accepted to be higher than that of a healthy person, the characteristics of the complicated sound waveforms make it difficult to identify abnormal sound signals from normal ones only based on the calculation of single complexity feature such as entropy. Two of the newly developed machine learning algorithms are based on entropy and other nonlinear analysis methods and have demonstrated high ability to discriminate variant types of sound signals with acceptable sensitivity and specificity. More algorithms such as neural network has been developed with outstanding performance in data analysis of various biological signals, such as detection of sleep apnea syndrome [45], arrhythmias [46] and sputum [47,48], and classification and evaluation of EEG waves [49]. Therefore, we suggest future investigation for establishing new diagnostic indices might be conducted with the help of novel deep learning algorithms.

Our review demonstrates the potential for entropy as a novel index of individualized diagnosis and prognosis prediction for asthma severity. However, before being fully developed as a valid tool in clinical practice, more investigations need to be done. First, the overall pattern of entropy changes should be explored in different statuses of diseases as we did in this review. There are other types of entropies, such as MSE and phase entropy computed using higher order spectra. We may need to explore the utility of these types of entropy in asthma related physiology signals. Second, analysis methods of entropy should be further optimized for different physiological signals. Per our review, practicable complexity analysis method has not been well developed for respiratory sound signals. For many signals, entropy results did not reach acceptable AUC values in every study group. Furthermore, the analytic results of entropy vary due to individual differences. Therefore, standardized value ranges of healthy subjects based on their vital biological information (sex, age, height, weight, etc.) are required as the foundation of the discrimination and classification of disease status. Besides, to evaluate the predictive power of entropies, we suggest longitudinal studies to reveal long-term time relations between the change of entropies and that of patients’ conditions.

In this paper, we systematically reviewed existing studies on entropy changes in asthma associated physiology signals and discussed their possible role in facilitating individualized medical sciences. Due to the limitation of our inquiry, certain articles might have been missed if they: (1) were published very recently; (2) were not included in the databases we searched; or (3) were written in a language other than English.

## 5. Conclusions

We reviewed existing research articles and summarized the entropy changes of physiology metrics associated with asthma. We found a significant correlation between complexity changes of physiological signals of asthmatic patients and the severity of asthma. For most signals we studied, such as airflow, HRV, COP and respiratory system impedance, their entropies decrease with the exacerbation of asthma. For respiratory sounds and airway resistance, their entropies increase in asthma patient. This might be explained by the unstableness of local respiratory tract and the increase of breath difficulty. ROC analysis demonstrated that entropies of several physiology signals have excellent sensitivity and specificity (AUC > 0.8 or even 0.9) when they were used to discriminate different severities of asthma. For future work, we suggest more clinical trials should be conducted to probe the entropy analysis for diagnosis and management of asthma. Besides, we also advise researchers to pay attention to the application of deep learning algorithms which has already shown extraordinary capability in physiological data analysis. The summarized result obtained through this review should give valuable guidance for further research on complexity patterns of physiological signals in asthma and provide basis for the use of entropy as a clinic indicator aiming for more individualized diagnosis and patient care.

## Figures and Tables

**Table 1:** GRADE analysis: applied entropy to physiologic parameters related with asthma.

OutcomeMeasure	N (Arms)	Risk of Bias	Limitation of Study	Inconsistency	Indirectness	Imprecision	Effect Size	Quality of Evidence
Airflow	128(3)	No	No obvious limitations	No	No indirectness	No	Significant	High
HRV	24(1)	No	No obvious limitations	No	No indirectness	No	Significant	High
entre of Pressure	39(1)	No	No obvious limitations	No	No serious indirectness	No	Significant	Moderate
Respiratory sound	51(3)	No	Limitation in study design and data collection	No	No serious indirectness	No	Significant	Low
Respiratory impedance	74(1)	No	No obvious limitations	No	No indirectness	No	Significant	High
Airway resistance	186(2)	No	Limitation in study design and data collection	No	No indirectness	No	Significant	Moderate

**Table 2:** Studies on Entropy Comparing Healthy subjects and Asthma Patients.

Physiologic Signals	Study (Year)	Study Type	Entropy Method	Location	Number of Subjects	Age in Years as Mean ± SD or Range	Gender Ratio (M/F)	Pulmonary Function	Entropy Result	AUC
Airflow	Veiga et al., 2010	Observational	ApEn	Brazil	Control 5 NE 5 Mild 5 Moderate 6 Severe 5	Control 47.6 ± 19.7 NE 33.2 ± 8.5 Mild 49.2 ± 14.7 Moderate 54.3 ± 7.8 Severe 61.4 ± 6.7	N/A	FVC, FEV_1_, FEF_25–75%_, FEV_1_/FVC, FEF/FVC	lower in asthmatic patients	No
	Veiga et al., 2011	Observational	ApEn	Brazil	Control 11 NE 11 Mild 14 Moderate 14 Severe 12	Control 54.4 ± 15.1 NE 34.9 ± 10.3 Mild 51.1 ± 13.5 Moderate 54.2 ± 10.7 Severe 60.5 ± 12.5	N/A	FVC, FEV_1_, FEF_25–75%_, FEV_1_/FVC, FEF/FVC	lower in asthmatic patients	Yes
	Raoufy et al., 2016	Observational	SampEn	Iran	Control 10 CAA 10 UAA 10 UNAA 10	Control 27.6 ± 5.3 CAA 30.8 ± 9.8 UAA 31.1 ± 7.2 UNAA 32.7 ± 8.1	N/A	N/A	lower in asthmatic subjects	Yes
HRV	Garcia-Araujo et al., 2014	Observational	ApEn SampEn Shannon	Brazil	Healthy 10 Asthma 14	Healthy 31 ± 8.7 Asthma 28 ± 8.5	Healthy: 10/0 Asthma: 11/3	FEV_1_, FVC, FEV_1_/FVC, VO_2_	lower in asthmatic patients during respiratory sinus arrhythmia maneuver	No
Center of pressure	Kuznetsov et al., 2014	Observational	SampEn	USA	Healthy 18 Asthma 21	Healthy 9.87 ± 2.77 Asthma 20.04 ± 1.85	Healthy: 3/15 Asthma: 6/15	N/A	lower in asthmatic patients	No
Respiratory Sound	Jin et al. 2008	Observational	SampEn	Singapore	Control 7 Asthma 7	N/A	N/A	N/A	SampEn is effective for wheeze detection.	No
	Aydore et al., 2009	Retrospective	Renyi	USA	7 (COPD & asthma)	50 ± 17	4/3	N/A	The Renyi entropy of wheeze signal has a uniform distribution	No
	Mondal et al., 2014	Retrospective	SampEn	India	Normal 10 Abnormal 20	N/A	N/A	N/A	higher in asthmatic subjects	No
Respiratory Impedance	Veiga et al., 2012	Observational	ApEn	Brazil	Control 12 NE 12 Mild 20 Moderate 18 Severe 12	Control 52.7 ± 16.4 NE 35.2 ± 9.9 Mild 51.8 ± 13.8 Moderate 53.2 ± 14.2 Severe 60.5 ± 12.5	N/A	FVC, FEV_1_, FEF_25–75%_, FEV_1_/FVC, FEF/FVC	higher in asthmatic patients	Yes
Airway Resistance	Gonem et al., 2012	Observational	SampEn	UK	Control: 30 GINA4: 33 GINA5: 33	Control: 47.0 ± 2.2 GINA4: 51.0 ± 2.3 GINA5: 56.5 ± 1.9	Control: 12/18 GINA4: 16/17 GINA5: 15/18	FEV_1_, FEV_1_/FVC	higher in asthmatic patients	No
	Umar et al., 2010	Observational	SampEn	UK	Control: 27 Asthma: 66	Control: 54.1 ± 1.4 Asthma: 48.4 ± 2.2	Control: 9/18 Asthma: 31/35	FEV_1_	higher in asthmatic patients	No

**Abbreviations:** NE: normal to exam; ILD: interstitial lung disease. FVC: Forced Vital Capacity; FEV_1_: Forced Expiratory Volume for the first second; FEF: Forced Expiratory Flow FEF_25_–___75%_: FEF between 25% and 75%; CAA: controlled atopic asthma; UAA: uncontrolled atopic asthma, UNAA: uncontrolled non-atopic asthma; VO_2_: maximal oxygen consumption; GINA: Global Initiative for Asthma; AUC: area under the Receiver Operating Characteristic curve of (ROC).
